# 4453 Public Health Ethics: Utilizing open education methods to foster interprofessional learning and practices

**DOI:** 10.1017/cts.2020.220

**Published:** 2020-07-29

**Authors:** Jeffrey S Farroni, Emma Tumilty

**Affiliations:** 1 UTMB Galveston; 2 School of Medicine, Deakin University Waurn Ponds

## Abstract

OBJECTIVES/GOALS: Innovative educational approaches and training modalities are important for training a diverse workforce in the authentic skills needed to advance all phases of clinical and translational research. Endeavors to study and develop policies that promote the translational science spectrum are steeped in value judgments. Learning how to navigate moral ambiguity and ethical reasoning enlightens our understanding of stakeholder obligations, roles and responsibilities. Ethics education can be challenging if learners are insufficiently engaged in the necessary critical reflection. In this course, decision-making in public health is informed through the analysis of the ethical issues, developing alternative courses of action and providing justification for actions taken in response to real-world dilemmas. The course is provided to students with a variety of backgrounds (science, health, policy) in a Master of Public Health degree program. Course objective were to: 1) Identify ethical issues in public health policy, practice, and research using appropriate concepts and terms; 2) Recognize the full spectrum of determinants of health and related information needed to resolve ethical conflicts in public health policy, practice, and research; 3) Present varied and complex information in written and oral formats; 4) Assess potential solutions to ethical conflicts in public health policy, practice, and research and 5) Decide ethical courses of action for public health policy, practice, and research. We adopted an open pedagogy as a guiding praxis to inform public health ethics discourse amongst our learners. In this way, learner agency was maximized to develop course materials within a generalized framework and shared with each other through the perspectives of each individual. The goal was to not only analyze complex ethical dimensions of public health issues but also gain insights into the disciplinary lenses of one’s peers. METHODS/STUDY POPULATION: Each week was divided into two sessions, a seminar and workshop. Course instructors introduce topics in a one-hour session and then allow students to decide what information is needed for a second session where the ethical issues of the topic will be discussed. Information-gathering tasks are then distributed amongst students in areas that are not their specialty, e.g. social history to be researched by learner with a biology background. The second session then involves the reporting back of background information by each student and a discussion of the ethical issues that arise. Through this process, the ability to communicate with others in different disciplines is supported, while exploring other disciplines and then engaging in ethical discussion and reasoning. Topics were introduced during the seminar session each week over the span of five weeks: 1) global public health, 2) disease prevention & control, 3) environmental & occupational public health, 4) resource allocation & priority setting and 5) research ethics. Learners were tasked with identifying the needed information to address the ethical, policy, and research aspects of the public health question(s) presented in these seminars. Students independently submitted resources they discovered to course instructors prior to the workshop. The *following* session began with a workshop where learners briefly presented their findings and deliberated on specific facets of the public health issue from that previous seminar while discussing a specific case. Students were assessed on their preparation (submission of identified resources), workshop presentation and participation. *Research Preparation:* In each seminar, the class decided what key information would be required to support the discussion at the workshop, which revolved around a relevant case study on that week’s topic. Course instructors facilitated the groups identification of material to be researched and the delegation of tasks within the group. Each student submitted a summary document (template provided) to course instructors prior to class for their area of research related to the case. *Research Presentation:* At the beginning of each workshop, each student was asked to present the research work to the rest of the class so that everyone has the same information for the case study discussion. These short (5-10 minutes) presentations followed the format of the preparation summary. *Participation/collaboration:* Both the seminar and the workshop asked students to be active learners within the class, participating in discussion, strategizing for information-gathering tasks, presenting researched material and arguments to others, and participating in case study discussion. Participation was assessed in relation to the value of the contributions made by students. RESULTS/ANTICIPATED RESULTS: The open pedagogy allowed the learners to construct the necessary materials to discuss issues with each other and develop not only a deeper understanding of the ethical dimension of public health issues but a shared understanding of each other’s disciplinary lenses. Course feedback was generally very positive, with learners either agreeing (33%) or strongly agreeing (67%) that the course was effective overall. In asking what learners liked best about the course, some indicated the “open pedagogy learning style” and “I liked the discussion format.” The positive comments mostly highlighted the discussion format. Areas for improvement noted by the learners included wanting “a longer course to cover more topics” and that the material was covered in “too short a time frame.” Other comments included that the course “was a bit disorganized” or that “the discussions were not very structured.” While the discussions by their very nature were unstructured, there is opportunity to refine this pedagogy to find right balance of learner agency. DISCUSSION/SIGNIFICANCE OF IMPACT: The goal of this teaching method was to empower the learner with the important critical thinking skills to navigate challenging ethical dilemmas in public health they may encounter in their careers. These skills include the identification of the ethical or moral conflict(s), collecting the necessary information to examine/resolve the dilemma, think creatively about the information that is unavailable and how to discuss/disseminate information to a broad constituency. This an educational model that is easily adaptable for learners working in other areas of the translational research spectrum, e.g. basic, pre-clinical, clinical and implementation sciences.

